# High On-Treatment Platelet Reactivity Affects the Extent of Ischemic Lesions in Stroke Patients Due to Large-Vessel Disease

**DOI:** 10.3390/jcm9010251

**Published:** 2020-01-17

**Authors:** Adam Wiśniewski, Joanna Sikora, Agata Sławińska, Karolina Filipska, Aleksandra Karczmarska-Wódzka, Zbigniew Serafin, Grzegorz Kozera

**Affiliations:** 1 Department of Neurology, Faculty of Medicine, Nicolaus Copernicus University in Toruń, Collegium Medicum in Bydgoszcz, 85-094 Bydgoszcz, Poland; 2 Experimental Biotechnology Research and Teaching Team, Department of Transplantology and General Surgery, Faculty of Medicine, Nicolaus Copernicus University in Toruń, Collegium Medicum in Bydgoszcz, 85-094 Bydgoszcz, Poland; joanna.sikora@cm.umk.pl (J.S.); akar@cm.umk.pl (A.K.-W.); 3 Department of Radiology and Diagnostic Imaging, Faculty of Medicine, Nicolaus Copernicus University in Toruń, Collegium Medicum in Bydgoszcz, 85-094 Bydgoszcz, Poland; agataslawinska@cm.umk.pl (A.S.); serafin@cm.umk.pl (Z.S.); 4 Department of Neurological and Neurosurgical Nursing, Faculty of Health Sciences, Nicolaus Copernicus University in Toruń, Collegium Medicum in Bydgoszcz, 85-821 Bydgoszcz, Poland; karolinafilipskakf@gmail.com; 5 Medical Simulation Centre, Faculty of Medicine, Medical University of Gdańsk, 80-210 Gdańsk, Poland; gkozera1@wp.pl

**Keywords:** platelet reactivity, ischemic stroke, aspirin resistance, infarction volume, multiplate

## Abstract

Background: Excessive platelet activation and aggregation plays an important role in the pathogenesis of ischemic stroke. Correlation between platelet reactivity and ischemic lesions in the brain shows contradictory results and there are not enough data about the potential role of stroke etiology and its relationships with chronic lesions. The aim of this study is to assess the relationship between platelet reactivity and the extent of ischemic lesions with the particular role of etiopathogenesis. Methods: The study involved 69 patients with ischemic stroke, including 20 patients with large-vessel disease and 49 patients with small-vessel disease. Evaluation of platelet reactivity was performed within 24 h after the onset of stroke using two aggregometric methods (impedance and optical), while ischemic volume measurement in the brain was performed using magnetic resonance imaging (in diffusion-weighted imaging (DWI) and fluid-attenuated inversion recovery (FLAIR) sequences) at day 2–5 after the onset of stroke. Results: In the large-vessel disease subgroup, a correlation was found between platelet reactivity and acute ischemic focus volume (correlation coefficient (R) = 0.6858 and *p* = 0.0068 for DWI; R = 0.6064 and *p* = 0.0215 for FLAIR). Aspirin-resistant subjects were significantly more likely to have a large ischemic focus (Odds Ratio (OR) = 45.00, 95% Confidence Interval (CI) = 1.49–135.36, *p* = 0.0285 for DWI; OR = 28.00, 95% CI = 1.35–58.59, *p* = 0.0312 for FLAIR) than aspirin-sensitive subjects with large-vessel disease. Conclusion: In patients with ischemic stroke due to large-vessel disease, high on-treatment platelet reactivity affects the extent of acute and chronic ischemic lesions.

## 1. Introduction

Stroke is one of the main causes of morbidity and long-term disability, and the second most frequent cause of death globally [1]. The updated definition of ischemic stroke by the American Heart Association/American Stroke Association (AHA/ASA) recognizes stroke as when clinical symptoms last less than 24 h and neuroimaging studies have demonstrated acute ischemic infarctions [2].

Currently, magnetic resonance (MR) imaging of the head is the gold standard in neuroimaging of the acute phase of ischemic stroke and aggregometry is the most popular methodology for the evaluation of platelet reactivity in the acute stage of stroke. In magnetic resonance (MR) imaging of the head, ischemic changes in the T2 and fluid-attenuated inversion recovery (FLAIR) sequences are described as hyper-intensive (i.e., with higher density than cerebral tissue). Additionally, imaging by means of the diffusion-weighted imaging (DWI) sequence, which is currently the most sensitive and the most specific method for imaging ischemic changes, shows the ischemic change just a few minutes after the onset of symptoms [3]. The ischemic focus in the DWI sequence is described as the area undergoing diffusion restriction. In order to assess the extent of an ischemic focus, its volume measurement via MR volumetry is used. Measurement of the volume of the area of interest is the most frequently performed technique, which consists of marking out the contours of the studied area in MR images, with further creation of a spatial structure and volume measures using dedicated software [4].

Platelet reactivity plays a role in the pathology of ischemic stroke, especially in nonembolic mechanisms (i.e., in patients with large and small vessels). Antiplatelet agents are the current standard in the secondary prevention of ischemic stroke, and European and American guidelines recommend acetylsalicylic acid (ASA) as the drug of first choice [5]. The efficacy of ASA can be decreased by a phenomenon called aspirin resistance [6]. Various platelet reactivity tests have been used to assess platelet activation and aggregation capacity, which allow for the assessment of platelet reactivity in response to the antiplatelet drug. High levels of platelet reactivity indicate a weak antiplatelet effect of the drug, which is estimated as laboratory resistance [7].

Previous studies reporting on the impact of aspirin resistance for ischemic lesion volumes in patients with stroke are scarce and have contradictory results. Some authors showed significant correlations between high platelet reactivity and large ischemic volumes in the brain [8,9], but there is still a lack of data about the potential roles of the etiology of strokes and relationships with the level of advancement of chronic vascular lesions in the brain. The aim of this study is to assess the platelet reactivity in patients in the acute phase of ischemic stroke and to investigate the relationships between platelet reactivity (estimated by impedance and optical aggregometry) and the volume of acute ischemic focus (volumetry in MR; DWI and FLAIR sequences) and chronic vascular changes (estimated using the Fazekas scale) in the brain with the particular role of cerebral ischemia etiopathogenesis.

## 2. Material and Methods

### 2.1. Research Subjects

The study was conducted in the Department of Neurology from February 2016 to December 2017 at the University Hospital No. 1 in Bydgoszcz. The prospective study included 69 subjects with diagnosis of ischemic stroke according to the updated definition of AHA/ASA. At the time of admission, all patients had been treated with ASA at a dose of 150 mg. Considering the etiopathogenesis of cerebral ischemia, patients were divided into groups with large-vessel disease (large artery atherosclerosis, LAA; *n* = 20 subjects) and small-vessel disease (SVD; *n* = 49 subjects). In the LAA group, significant atherosclerotic changes in the internal carotid artery was the cause of stroke; in the SVD group, lacunar changes in deep structures of the brain tissue were responsible for stroke symptoms. The LAA group included patients with a carotid artery stenosis >50% on the side, corresponding to the symptoms of stroke conforming to a Doppler ultrasound examination of the carotid arteries. The SVD group included patients with no significant hemodynamic stenoses of large precranial vessels or cardiogenic embolic backgrounds, in which neuroimaging confirmed the presence of a lacunar focus and revealed chronic vascular changes with a typical location and morphology for small-vessel disease (i.e., subcortical lesions, periventricular lesions, and leukoaraiosis features) [10]. The following exclusion criteria were used: lack of informed consent for the patient to participate in the study (quantitative disturbances of consciousness or aphasia), cardioembolic etiology of stroke (documented atrial fibrillation, dilated cardiomyopathy, thrombus in the heart cavities), chronic inflammatory processes (chronic lower limb ischemia or chronic venous thrombosis of the lower limbs), stroke or transient ischemic attack (TIA) in the previous 2 years, documented neoplasms, taking acetylsalicylic acid before admission to the clinic, past significant bleeding in the previous 2 years (e.g., gastrointestinal bleeding), level of hemoglobin < 9 g/dL, thrombocytopenia < 100 thousands/uL, or value of hematocrit <35%. The general characteristics of the studied population and a comparison of the groups of patients with large-vessel disease and small-vessel disease are shown in Table 1.

All subjects signed an informed consent form to participate in the study and read the protocol. The study protocol received a positive opinion from the Bioethics Committee of the Nicolaus Copernicus University in Torun at Collegium Medicum of Ludwik Rydygier in Bydgoszcz (KB number 73/2016).

### 2.2. Platelet Reactivity Research Methodology

The platelet reactivity assessment was performed by optical aggregometry and impedance aggregometry in the Laboratory of Biotechnology at Collegium Medicum in Bydgoszcz. Blood tests were performed at a similar time of day (10:00–12:00) in the first 24 h of onset of stroke symptoms. The optical aggregometry test was performed using a Chrono-Log aggregometer with the Agrolink software for Windows (Havertown, PA, USA). Using the apparatus, the ability of platelet aggregation in platelet-rich plasma was evaluated in vitro by measuring the change in transmission of light passing through the platelet suspension in response to the addition of a platelet agonist. After the activation of platelets by the agonist, the plasma suspension cleared before the formation of platelet aggregates; thus, the amount of light absorbed by the photometer increased. Arachidonic acid was used as the agonist, which activated the cyclo-oxygenase No 1 (COX-1) enzyme, the point of action of acetylsalicylic acid. The aggregometer analyzed changes in transmitted light in percentage and automatically converted this to a graphical curve that reflected changes in light transmission as a result of platelet aggregation. The most important element visually was the area under the curve (AUC), which depicted the amount of light absorbed over time. An average increase in absorbance above 20% (or AUC units > 115) was considered as ASA resistance, equivalent to a high on-treatment platelet reactivity induced by arachidonic acid. Approximately 9.4 mL of blood was collected in the morning on an empty stomach from the veins of the forearm into tubes with an admixture of 3.2% sodium citrate at a ratio of 1:9. Tests were performed up to 2 h after blood collection. Samples were centrifuged at 100 *g* for 10 min at room temperature to obtain platelet-rich plasma (PRP), and at 2400 *g* for 20 min at a temperature of 40 °C to obtain platelet-poor plasma (PPP). The number of platelets was calculated before the activation was assessed and the number was corrected to 250,000/µL. The percentage of aggregation induced with arachidonic acid in the final concentration of 0.5 µM was assessed [11]. The evaluation of optical aggregometry was performed in 43 out of 69 subjects due to aggregometer failure.

**Figure 1:**
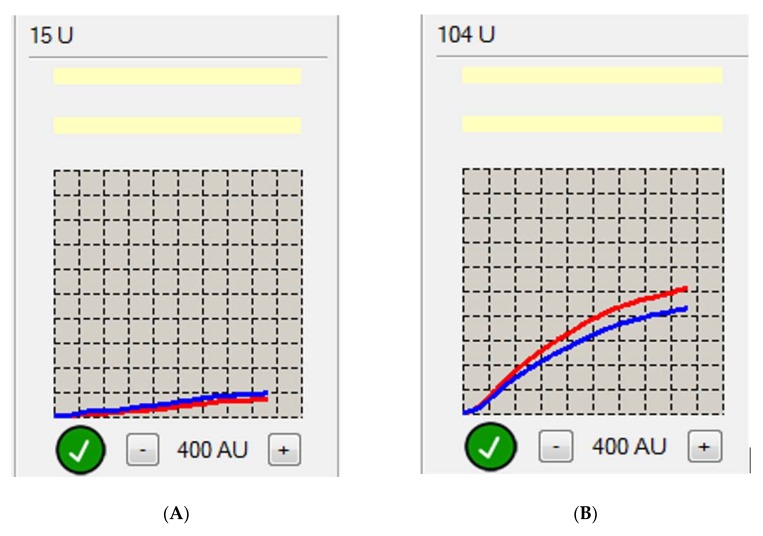
Curves of platelet reactivity assessed by impedance aggregometry: (**A**) Aspirin (ASA)-sensitive subject with 15 area under the curve (AUC) (**B**) ASA-resistant subject with 104 AUC. First measurement- blue line, second measurement- red line.

The Multiplate–Dynabyte multichannel platelet function analyzer (Roche Diagnostics, France) was used to perform impedance aggregometry. The Multiplate system, based on the impedance aggregation method in whole blood, used the so-called multiple electrode aggregation (i.e., a double marking was performed during each measurement). In this study, the acetylsalicylic acid platelet inhibitor (ASPI) test (measuring aggregation dependent on cyclo-oxygenase) was applied with arachidonic acid as a platelet activator. Two electrodes were immersed in a blood sample with a stirrer and the blood platelets aggregated after the addition of the agonist, causing them to accumulate onto the electrodes, resulting in a change in electrical resistance (impedance) between them. Impedance changes were then converted into a graphical model depicting the change in platelet aggregation over time. The AUC parameter was reported as the final result of the determination. The results above 40 AUC units were considered to be high on-treatment platelet reactivity induced by arachidonic acid, similar to ASA resistance (see Figure 1). Subjects with AUC values under 30 were treated as sensitive to ASA, while from 30–40 was considered as mild sensitivity to ASA. From each patient, approximately 2.6 mL of blood was collected in the morning from the forearm veins into a Sarstedt r-hirudin-type tube. After collection, the blood was kept for at least 30 min but for no more than 2 h. At first, 300 µL of sodium chloride was prepared and heated up to 37 degrees C, then 300 µL of whole blood was prepared in a special Dynabyte disposable test chamber with a magnetic stirrer, placed into four consecutive measuring stations, and connected to the device. Then, after 3 min of incubation, 20 µL of the reagent (arachidonic acid) was added. The aggregation test was assessed for 6 min, and the final result (in the form of AUC units) was the mean of the two measurements in the form of a curve [12]. The evaluation of impedance aggregometry with the use of the Multiplate system was performed in all 69 subjects.

### 2.3. Doppler Examination of the Carotid Arteries

Doppler examination of the carotid arteries was performed in all subjects within 5 days after the onset of symptoms of cerebral ischemia using a MyLab C Class (ESAOTE, Genoa, Italy) in the Doppler Laboratory of the Neurological Department of Dr A. Jurasz University Hospital No. 1 in Bydgoszcz. The study was performed based on the guidelines of the American Neurological Society [13].

### 2.4. Magnetic Resonance Imaging of the Head with Volumetric Evaluation

**Figure 2:**
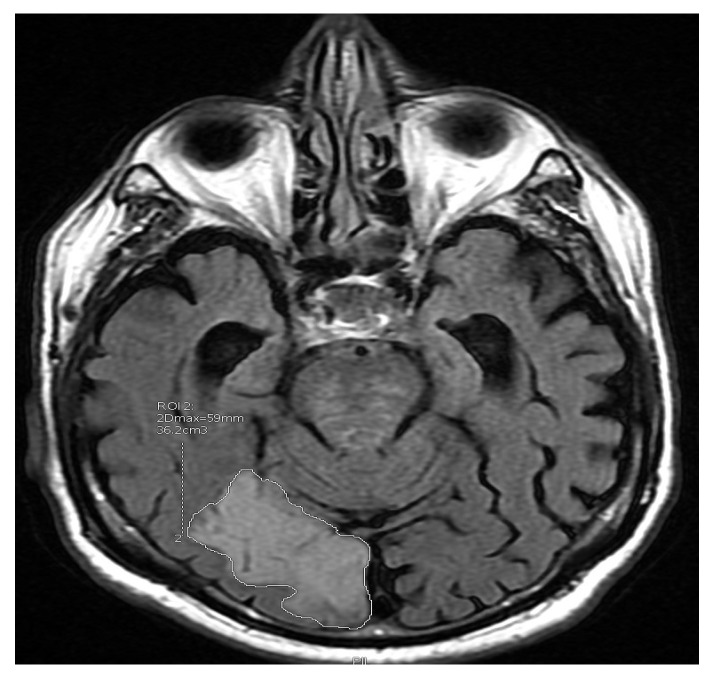
Volumetric evaluation of ischemic focus size in magnetic resonance (MR) in fluid-attenuated inversion recovery (FLAIR) sequence.

Magnetic resonance imaging was performed during hospitalization in the Department of Neurology within 2–5 days from the onset of symptoms of cerebral ischemia in the Magnetic Resonance Laboratory of the Department of Radiology, Dr A. Jurasz University Hospital No. 1, Bydgoszcz, with a 1,5 T Signa HDX apparatus (G.E. Healthcare, Chicago, IL, USA). The study was performed without a contrast agent following the so-called “stroke protocol”, evaluating brain images in T1, T2, FLAIR, and DWI sequences. The volumetric evaluation (shown in Figure 2) in the FLAIR and DWI sequences was estimated on a “layer-by-layer” basis, using the G.E. Advanced Workstation 4.6 by an experienced radiologist and given in millimeters (1 mL = 1 cm^3^) [4]. Based on the volumetry method of the subjects with stroke, they were divided into patients with a large ischemic focus (> 2.5 mL in DWI and > 3.5 mL in FLAIR) and a small ischemic focus in the brain.

The extent and the degree of chronic ischemic changes in periventricular white matter was estimated by the periventricular white matter (PVWM) Fazekas scale, and in deep white matter by the deep white matter (DWM) Fazekas scale. A total of 14 subjects with LAA and 43 with SVD underwent MRI examination.

### 2.5. Statistical Evaluation Methods

Nonparametric tests due to the incompatibility of the distribution of features with the normal distribution were used: the chi-square test (relations between categorized variables), Spearman’s rank correlation test (relations between variables), and the Mann–Whitney U test (relations between binary and continuous variables). Logistic regression analysis was performed to estimate the probability of a large ischemic outbreak, comparing ASA-sensitive and -resistant subjects. The significance level of *p* < 0.05 was considered to be statistically significant. Statistical evaluation was performed with STATISTICA software (version 13.1, Dell Inc., Round Rock, TX, USA). 

## 3. Results

### 3.1. All Subjects

The median of ischemic focus volume was 1.81 mL (minimum 0.09 mL, maximum 10.1 mL) in the DWI sequence and 2.41 mL (minimum 0.18 mL, maximum 31.8 mL) in the FLAIR sequence. In patients with stroke, there were no statistically significant correlations between platelet reactivity and the size of the ischemic lesion, as assessed both in the DWI (with the Multiplate method, R = 0.0700 and *p* = 0.6243; with optical aggregometry, R = −0.0670 and *p* = 0.7064), and FLAIR (with the Multiplate method, R = 0.083 and *p* = 0.5648; with optical aggregometry, R = −0.0500 and *p* = 0.7783) sequences. In ASA-resistant patients (by Multiplate), the median ischemic focal size did not differ significantly from the median ischemic focus size in ASA-sensitive patients in both DWI and FLAIR sequences (DWI median: 3.94 versus 1.305 mL, *p* = 0.4755; FLAIR median: 4.88 versus 2.09 mL, *p* = 0.5241). There were also no significant differences in platelet reactivity between the groups of patients with large and small ischemic focuses (DWI sequence median: 31.5 AUC versus 23 AUC, *p* = 0.3226; FLAIR sequence median: 33.5 AUC versus 23 AUC, *p* = 0.3559). No significant correlations between platelet reactivity and the extent and degree of chronic ischemic lesions in periventricular or deep white matter were found either (PVWM Fazekas R = 0.0067, *p* = 0.9667; DWM Fazekas R = 0.00547, *p* = 0.9742).

### 3.2. Subgroups of LAA and SVD Subjects

**Figure 3:**
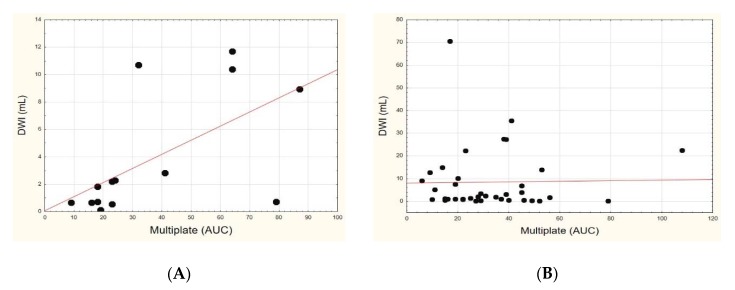
Correlation between platelet reactivity as assessed by Multiplate method (in area under the curve (AUC) units) with the ischemic focus size assessed in diffusion-weighted imaging (DWI) (in mL) sequence in the large-artery atherosclerosis subgroup (**A**) and small-vessel disease subgroup (**B**).

In the subgroup of patients with LAA, there was a significant positive correlation between the platelet reactivity in the impedance aggregometry method and the ischemic focal size, as assessed both in the DWI (Figure 3A) and FLAIR sequences (R = 0.6858 and *p* = 0.0068 for DWI; R = 0.6064 and *p* = 0.0215 for FLAIR). There was no correlation between platelet reactivity and ischemic focus size in the SVD subgroup (R = −0.1107 and *p* = 0.5139 for DWI; R = −0.1055 and *p* = 0.5342 for FLAIR). 

**Figure 4:**
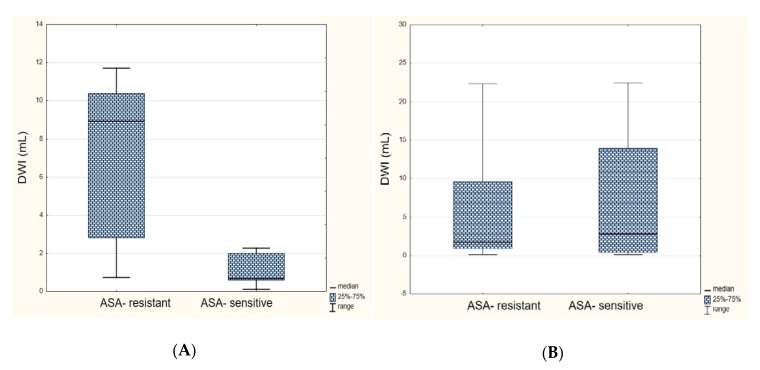
Comparison of ischemic focus size (in mL) in the diffusion-weighted imaging (DWI) sequence in the group of patients resistant and sensitive to Aspirin (ASA) in the large artery atherosclerosis subgroup (**A**) and the small-vessel disease subgroup (**B**).

**Figure 5:**
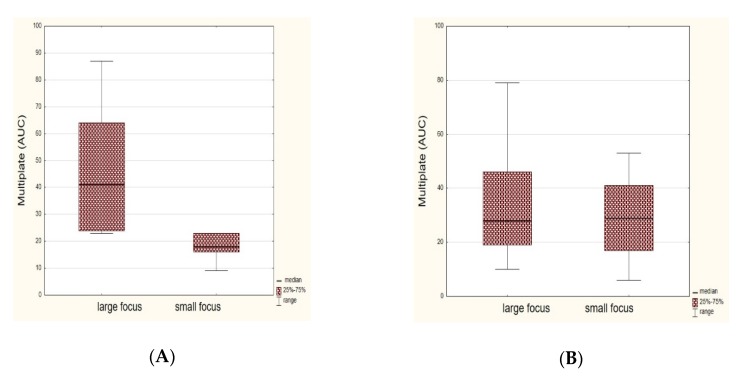
Comparison of the platelet reactivity (in area under the curve (AUC) units) assessed by the Multiplate method in subjects with a large and small ischemic focus in the diffusion-weighted imaging (DWI) sequence, in both the large-artery atherosclerosis subgroup (**A**) and the small-vessel disease subgroup (**B**).

There were no significant correlations between platelet reactivity and the ischemic volume in either subgroups based on the optical aggregometry method. In the subgroup with LAA, ASA-resistant patients (assessed by Multiplate) had a significantly larger median volume of ischemic focus, both in DWI (Figure 4A) and FLAIR, as compared to patients sensitive to ASA (DWI median: 8.93 versus 0.68 mL, *p* = 0.0157; FLAIR median: 11.0 versus 1.21 mL, *p* = 0.0338). In the SVD subgroup, no similar significant relationships were found (*p* = 0.8259 for DWI; *p* = 0.7084 for FLAIR). In the subgroup of subjects with LAA, the median platelet reactivity assessed by the Multiplate method was higher in the subgroup of patients with a large ischemic focus in the DWI sequence than in the group with small ischemic focus (median: 41 versus 18 AUC, *p* = 0.0253) (Figure 5A). There were no significant relationships in the FLAIR sequence (median: 41 versus 19 AUC, *p* = 0.0639) in the LAA subgroup and in SVD subjects, either with the impedance or optical aggregometry methods. 

In the LAA subgroup, a significant correlation between platelet reactivity in the Multiplate method and the extent and degree of chronic ischemic lesions was demonstrated, both in periventricular and deep white matter (PVWM Fazekas R = 0.5569, *p* = 0.0386; DWM Fazekas R = 0.5590, *p* = 0.0377). In the SVD subgroup, no significant relationships in this subject were found (PVWM Fazekas R = −0.1613, *p* = 0.3403; DWM Fazekas R = −0.2479, *p* = 0.1356).

Logistic regression analysis showed that in the subgroup of patients with LAA, ASA-resistant patients (by Multiplate) had a significantly higher probability of a large ischemic focus than patients who were sensitive to ASA (OR = 45.00, 95% CI 1.49–135.36; *p* = 0.0285 for DWI and OR = 28.00, 95% CI 1.35–58.59; *p* = 0.0312 for FLAIR). There were no similar significant relationships in the subgroup of patients with SVD (OR = 1.0, 95% CI 0.22–4.56, *p* = 1.0 for DWI and FLAIR) and in the whole group of patients with stroke (OR = 2.55, 95% CI 0.70–9.31, *p* = 0.1566 for DWI; OR = 2.70, 95% CI 0.74–9.81, *p* = 0.1313 for FLAIR).

## 4. Discussion

It has been proven that ASA-resistant subjects are at significantly higher risk of stroke recurrence, heart attack, and death due to vascular disease. Furthermore, resistance to ASA has been associated with the male gender and smoking [14,15]. The spread of aspirin resistance in subjects with stroke has been estimated at 5–65% [16]. In our own research, based on the method of impedance aggregometry, ASA resistance was estimated among patients with stroke at 31.8% and at 7% in the method of optical aggregometry, which coincides with the data from the literature. Considering the increasing incidence of stroke (20% increase predicted by 2030 as a result of population aging) [17], it is necessary to study the impact of ASA resistance on the size of the ischemic focus. 

Our study demonstrated the significant effect of etiopathogenesis of ischemic stroke on the relationship between platelet reactivity and the size of an ischemic focus. There was a highly positive correlation observed between platelet reactivity and size of the acute ischemic focus in the subgroup of patients with large artery atherosclerosis. Moreover, it was noted that aspirin-resistant patients with carotid artery pathology had a larger stroke volume than patients who were sensitive to ASA, and at the same time, that the large ischemic focus in DWI was related to higher platelet reactivity. Additionally, there was a highly positive correlation observed with the extent and the degree of chronic ischemic changes in white matter in large artery atherosclerosis subgroup. This should be treated as novelty in this field, because the literature does not address the impact of stroke etiopathogenesis on the correlation of platelet reactivity and the size of the acute ischemic focus. To the best of our knowledge, we are first to underline the role of high on-treatment platelet reactivity for the extent of chronic vascular lesions in the brain.

However, the results presented in this study did not lead to finding any significant relationship between platelet reactivity and the size of an ischemic focus in the whole group of patients with stroke. Oh et al. [8] documented that patients with ASA resistance had a statistically significantly larger ischemic focus size, as assessed in MR-DWI (median 5.4 mL versus 1.7 mL), than the group of patients sensitive to ASA. Similar results were presented by Cheng et al. [9], whose differences between the ASA-resistant and -sensitive groups were even more significant (13.21 mL versus 4.26 mL). In addition, they showed a significant correlation between the reactivity of the platelets and the size of the ischemic focus (R = 0.63). In turn, Agayeva et al. [18] and El-Mitwalli et al. [19] did not show the influence of platelet reactivity on the ischemic focus size, as assessed by MR-DWI in the brain. Discrepancies with the results of Oh et al. [8] and Cheng et al. [9] may have resulted from several important elements. First of all, the methodology was as follows: all of the above tests were performed based on turbidimetry aggregometry, which as demonstrated by Larsen et al. [20] and Ko et al. [21], poorly correlates with the Multiplate method. Secondly, other publications have studied patients with all types of strokes (including embolic). Due to other mechanisms of activation of the coagulation cascade, the limited therapeutic effect of ASA, and the possible use of low molecular weight heparin therapy in a treatment dose from day 1 of the stroke, patients with embolic stroke were excluded from this study. The aim of this procedure was also to unify the group of subjects to only those in which the pathology of platelet function is of the greatest importance in the etiopathogenesis of stroke (thrombotic mechanism) and in which the benefits of ASA therapy are the greatest. Thirdly, in all of the above studies, the patients took acetylsalicylic acid at least 7 days before the onset of the stroke. It is worth mentioning that the population of patients studied in this study included only those who had not taken acetylsalicylic acid before the onset of stroke. The aim of this procedure was not only to unify the study group, but also to maintain the right proportions in relation to the epidemiology of stroke, as the data from the literature showed that the vast majority of patients, at the same time of developing stroke, did not previously take ASA [22]. Data from the literature provides information that the fact of taking ASA before stroke is relevant to the results obtained. Rosafio et al. [23] and Sabra et al. [24] showed significant differences in platelet reactivity between a group that took ASA for the first time in the acute phase of stroke and a group that had received ASA prior to stroke. In addition, some authors have shown that taking ASA before has a potential effect on the size of an ischemic focus [25]. It is worth mentioning that Cheng et al. [9] always evaluated the size of the ischemic focus size in DWI within the first day of stroke, and Oh et al. [8] within 48 h. In this study, the MR examination was performed up to 5 days after, usually on the 3rd or 4th day, but never within the first 24 h. It cannot be excluded that these time discrepancies, which have no effect on the size of the ischemic focus, may have contributed to the contradictory results. During the first few hours of stroke, the area of ischemia in DWI may include—aside from the area of irreversibly damaged tissue—the penumbra area (i.e., the area at risk of infarction progression), which after several hours (sometimes even after 24 h) may be remitted due to reperfusion [26,27].

Theoretically, greater activation and aggregation of platelets in the presence of the platelet inhibitory effect by ASA should create microaggregates from platelets and platelet–thrombin complexes, and the release of pro-spasmodic factors, leading to a larger vascular plug formation, occlusion of the larger vessel, and finally leading to a larger area of cerebral ischemia [28]. However, the authors of this study believe that this mechanism may be important, but only at the level of arteries of a larger caliber. In patients with small-vessel disease, small arterioles and capillaries, which are initially narrowed by advanced atherosclerotic processes, ultimately close when the thrombus is formed, leading to a relatively small ischemic outbreak. In this case, excessive activation of platelets, which may lead to the closure of several additional fine arterioles in the areas, does not significantly affect the size of the ischemic focus. Nevertheless, in the case of patients with large-vessel disease, excessive activation and platelet aggregation may significantly affect the closure of the larger vessel, which will lead to a globally larger ischemic area from the larger vascularization basin. This relationship was demonstrated in this analysis, finding a high correlation between the platelet reactivity and the size of the ischemic focus—the higher the activity of platelets in patients with a larger vessel closure, the larger the ischemic focus in the brain. However, it is worth noting that this study was dominated by patients with small-vessel disease and globally small ischemic foci, in which no correlation with platelet reactivity was demonstrated. This can be used to explain the fact that in this study, patients with ASA resistance and higher platelet reactivity obtained larger median sizes of ischemic focal size in all analyses; however, these values were not significant for the entire stroke group, even at the level of statistical tendency. 

The obtained results of this study suggest that large artery atherosclerosis appears to be an important factor in activating platelets. Independent authors have demonstrated, by means of various methods of assessing platelet reactivity, higher platelet activation in patients with the large extracranial vessel disease [29,30,31,32]. Moreover, the beneficial role of surgical interventions on the carotid artery in improving platelet function has also been emphasized. Kinsella et al. [33] showed that high platelet reactivity was observed in patients after stroke in the course of carotid artery stenting after carotid stenting decreased below the level found in asymptomatic patients, who were treated only with the antiplatelet drug. The above results undoubtedly prove the important role of platelets in the pathogenesis of stroke in patients with carotid artery stenosis, which additionally, in the light of the results of this analysis, puts the group of patients with large artery atherosclerosis as special beneficiaries of optimal antiplatelet therapy. The authors of this study also believe that the effects of etiopathogenesis of ischemic stroke may also result from a pathomorphic basis. In patients with LAA, the classic atherosclerotic process dominates, in which the role of platelet activation is indisputable and primary; in patients with small-vessel disease (especially in small arteries under 200 µm), fibrosis, glazing, calcification, and lypohialinose processes prevail, which pathomorphologically different than the classical atherosclerotic process and in which the role of platelets is limited and marginal [34]. These pathomorphological differences can undoubtedly result in the relationship between excessive activation of platelets and a larger area of cerebral ischemia.

However, the current study has its limitations: The size of the studied population was moderate, but seemed sufficient to make conclusions. Additional studies on larger populations of subjects are needed to confirm our theories. Due to the failure of the optical aggregometer, most conclusions were based only on the results of impedance aggregometry (Multiplate analyzer), which similar to other platelet reactivity tests, is poorly standardized with agreed resistance criteria [35]. The main limitation of our study is that it is a one-time, single measurement of platelet activation at different times after the onset of stroke symptoms and after the dose of acetylsalicylic acid, which may be insufficient to properly assess the relationships between ASA resistance and the size of an ischemic focus. An optimal approach seems to be the sequential determination of platelet reactivity on successive days [36,37,38]. Another limitation is poor correlation between biochemical (laboratory) and clinical ASA resistance [39]. Thus, the results of the ex vivo platelet reactivity tests do not always translate to the therapeutic effect of the antiplatelet drug. Agayeva et al. [40] suggested that the reason for this may be the heterogeneity of the pathophysiological basis of ischemic brain stroke. One should also remember the anti-inflammatory and neuroprotective activities of ASA, which take place by mechanisms independent of the platelets [41]. Another limitation seems to be the lack of assessment of the potential impacts of other drugs used by stroke patients (such as statins, which are routinely recommended in the secondary prevention of stroke) and the ASA dose itself on platelet reactivity. Due to the low detectability of atrial fibrillation as a cause of stroke, the authors of the study are also uncertain whether there was a small percentage of patients in the study population with embolic cerebral ischemia. In turn, for formal reasons (i.e., conscious consent for the study given to the Bioethics Committee), the analysis did not include patients with a severe neurological deficit (e.g., patients with impaired consciousness); hence, the study does not include a full cross-section of patients with stroke, but only patients with milder clinical conditions.

## 5. Conclusions

In stroke patients with large-vessel disease, high on-treatment platelet reactivity is associated with a larger extent of acute ischemic focus and chronic ischemic lesions in the brain. The important roles of etiopathogenesis of stroke in the occurrence of significant relationships between platelet reactivity and the extent of both acute and chronic brain lesions have been highlighted. Further research is needed to determine the optimal therapeutic strategies in stroke due to large-vessel disease coexisting with high on-treatment platelet reactivity.

## Figures and Tables

**Table 1:** Comparison of the selected risk factors and biochemical parameters obtained in subjects with stroke in both etiological subgroups of cerebral ischemia.

Parameter	LAA *N* = 20	SVD *N* = 49	*p* Values
Age median (range) *	67 (45–85)	68 (40–89)	0.7761
Sex, male, *N* (%) **	14 (70%)	21 (42.9%)	**0.0408**
Hypertension, *N* (%) **	17 (85%)	44 (89.7%)	0.6822
Diabetes, *N* (%) **	8 (40%)	17 (34.7%)	0.7842
Hyperlipidemia, *N* (%) **	10 (50%)	18 (36.7%)	0.4185
Smoking, *N* (%) **	11 (55%)	13 (26.5%)	**0.0299**
Ischemic heart disease, *N* (%) **	3 (15.0%)	6 (12.2%)	0.7120
CRP (mg/L) median (range) *	4.90 (0.43–30.45)	4,79 (0.36–29.15)	0.7660
HbA1c (%) median (range) *	5.9 (5.2–10.04)	5.6 (5.0–9.8)	0.2313
Homocystein (µmolµ/L) median (range) *	10.67 (5.1–30.92)	10.87 (3.52–48.6)	0.6153
Fibrinogen (mg/dL) median (range) *	312.5 (234–590)	300 (369–540)	0.2992
Obesity, *N* (%) **	12 (60%)	36 (73.47%)	0.2699
The volume of ischemic focus DWI (cm^3^) median (range) *	2.23 (0.12–10.3)	0.89 (0.09–1.5)	0.0694
The volume of ischemic focus FLAIR (cm^3^) Median (range) *	3.46 (0.35–31.8)	0.99 (0.18–1.71)	0.0846
Platelet reactivity: optical aggregometry (AUC) median (range) *	17.1 (0–208.6)	20.4 (0–154.2)	0.7147
Platelet reactivity: impedance aggregometry (AUC) median (range) *	42 (9–101)	27.5 (6–108)	0.0622
Resistance to ASA (multiplate), % **	52.3%	22.9%	**0.0286**

* U Mann-Whitney test; ** chi square test. Note: FLAIR = fluid-attenuated inversion recovery; ASA = acetylsalicylic acid; AUC = area under the curve; DWI- diffusion-weighted imaging; CRP = C-reactive protein; HbA1c- glycated hemoglobin; LAA = large artery atherosclerosis; SVD = small-vessel disease; *N* = number of subjects, bold font = statistically significant p Values.
